# A case of Axenfeld–Rieger syndrome with neuroradiological abnormalities

**DOI:** 10.1016/j.radcr.2025.06.072

**Published:** 2025-07-23

**Authors:** Yu Ohkubo, Takaki Hayashi, Kohmei Ida, Eiji Nakano, Akiyoshi Hamada, Tomohiko Higashida, Yasumasa Asakawa, Kohki Yoshikawa, Tokuzo Yokokawa, Masao Tago

**Affiliations:** a Department of Radiology, Teikyo University Mizonokuchi Hospital, Kawasaki, Japan; b Department of Pediatrics, Teikyo University Mizonokuchi Hospital, Kawasaki, Japan

**Keywords:** Axenfeld–Rieger syndrome, FOXC1, Chromosome 6p25, White matter lesion, Cerebral small vascular disease

## Abstract

Axenfeld-Rieger syndrome is a rare autosomal dominant disorder characterized primarily by developmental anomalies of the anterior segment of the eye and systemic manifestations, including craniofacial abnormalities, dental anomalies, and neurological involvement. Although genetic mutations in the forkhead box C1 (FOXC1) or paired-like homeodomain transcription factor 2 (PITX2) have been implicated, the full extent of associated neurological features remains underexplored. We present the case of a 2-year-old boy diagnosed with Axenfeld-Rieger syndrome who exhibited mild facial dysmorphism and required surgical intervention for glaucoma. Genetic testing identified a FOXC1 mutation, and family history revealed that his father and paternal grandfather were also affected. Brain magnetic resonance imaging scans revealed periventricular white matter lesions, dilated perivascular spaces, and vertebrobasilar artery dolichoectasia. This case underscores the potential for significant neurological findings in patients with Axenfeld-Rieger syndrome and highlights the clinical value of comprehensive neuroradiological evaluation in such cases.

## Introduction

Axenfeld–Rieger syndrome (ARS) is an autosomal dominant genetic disorder characterized by anterior segment abnormalities of the eye and associated with various other features spanning multiple bodily systems. Its estimated prevalence is 1 in 50,000–100,000 newborns [1]. Mutations in the transcription factors forkhead box C1 (FOXC1) or paired-like homeodomain transcription factor 2 (PITX2), on chromosomes 6p25 and 4q25, respectively, are the most widely researched genetic manifestations of this syndrome. Nevertheless, the specific genetic mutations responsible for 60% of ARS cases remain unidentified [2].

ARS-associated ocular features include posterior embryotoxin (Schwalbe ring thickening and anterior displacement), iris hypoplasia, corectopia (displaced pupil), pseudopolycoria (additional pupillary opening), and iridocorneal adhesions [3]. Secondary glaucoma is often the most severe ARS consequence, potentially inducing permanent blindness within several years. ARS could also cause non-ocular systemic defects such as facial dysmorphisms (e.g., prominent forehead, hypertelorism, telecanthus, maxillary hypoplasia, and flattened mid-face), dental (e.g., microdontia, hypodontia, and anodontia) and cardiovascular anomalies, hearing impairment, and developmental delays [1]. ARS-related neurological manifestations typically include abnormalities of the sella bone, hydrocephalus, and white matter-related variations. ARS-associated neurovascular anomalies have also been documented, including stroke and cerebral small-vessel disease (CSVD).

In this study, we describe an ARS case associated with neuroradiological findings.

## Case report

A 2-year-old boy presented at our center with psychomotor retardation. He was born via forceps-assisted delivery at 36 weeks of gestation. He displayed a birth weight of 3144 g, an Apgar score of 8/9, and an unremarkable pre-perinatal history. His craniofacial features included orbital hypertelorism, flat nasal bridge, and low-set ears. Cranial ultrasonography revealed no corpus callosum agenesis, intracranial hemorrhage, or ventriculomegaly. The periventricular echodensity grade was 1. He was able to hold his head up at 4–5 months, sit upright at 8–9 months, pronounce his first word at 12 months, walk unassisted at 19 months, and speak two-word sentences at 31 months.

An ophthalmological assessment at 1 month of age revealed corneal opacity and increased intraocular pressure. The patient subsequently underwent glaucoma surgery at 3 months of age.

The family history of the patient was significant, as his father exhibited an atrial septal defect, glaucoma at 2 years of age, bilateral auditory disturbances in his teens, and cerebral infarction at 18 years of age. Moreover, his paternal grandfather suffered from glaucoma and auditory disturbances as a teenager.

**Fig. 1:**
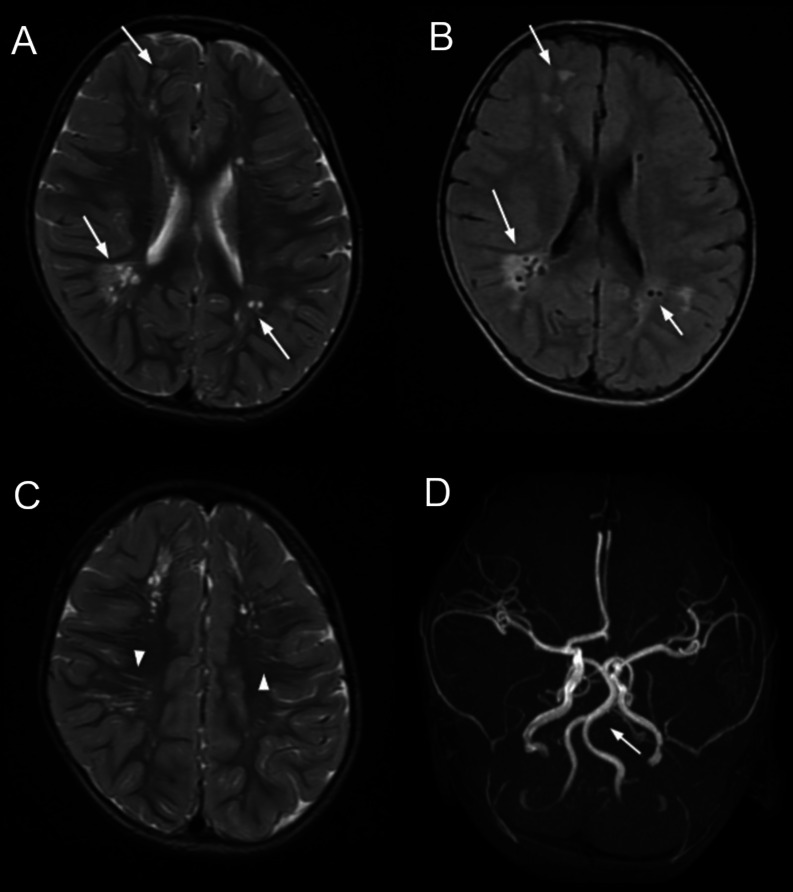
Magnetic resonance imaging of the patient at 2 years of age. On the axial T2-weighted (A) and fluid-attenuated inversion recovery (B) images, multifocal cerebral white matter lesions are visible (arrows). The T2-weighted image shows hyperintense thin stripes suggesting dilated perivascular spaces (C, arrowheads). Magnetic resonance angiography revealed vertebrobasilar artery dolichoectasia (D, arrow).

The patient underwent magnetic resonance imaging (MRI) of the brain at 2 years of age to investigate the cause of his developmental delay. White matter lesions were detected with hypointensity on T1-weighted imaging, hyperintensity on T2-weighted imaging, and a peripheral hyperintense rim on fluid-attenuated inversion recovery imaging (Fig. 1A and B). Furthermore, radiating thin stripes with high signal intensities were observed on T2-weighted imaging, suggesting increased perivascular spaces (Fig. 1C). Magnetic resonance angiography revealed dolichoectasia of the vertebrobasilar artery (Fig. 1D). No evidence of any recent hemorrhage was detected. The corpus callosum of the patient was normal, and no ventriculomegaly was detected.

Genetic testing revealed a *FOXC1* gene mutation, c.240del(p.Y81Ifs*21), consistent with an ARS diagnosis.

## Discussion

ARS is a rare hereditary disorder characterized by multisystemic developmental abnormalities.

The reported neuroradiological feature prevalence in ARS cases varies in the literature. According to a systematic review, the most common ARS-associated neurological findings are white matter abnormalities, such as white matter hyperintensities, leukoencephalopathy, and periventricular white matter lesions, appearing in 41.3% of the cases. The second most common observation is the Dandy-Walker complex. Corpus callosum agenesis, ventriculomegaly, hydrocephalus, and dilated perivascular spaces could also be observed in several cases [2]. Another case series described vertebrobasilar artery dolichoectasia as the most common observation, followed by white matter hyperintensities, hemispheric or global cerebellar hypoplasia, corpus callosum thinning, and ventriculomegaly [3]. In the hereby-presented case, white matter abnormalities, dilated perivascular spaces, and vertebrobasilar artery dolichoectasia were observed, whereas the corpus callosum of the patient seemed normal and no ventriculomegaly was evident.

Previous studies suggest that FOXC1 is crucial for heart, kidney, eye, and brain development. French et al. published that FOXC1 mutations could induce CSVD, white matter hyperintensities, dilated perivascular spaces, microbleeds, and lacunar infarcts, suggesting that FOXC1 contributes to vascular stability [4]. Whitesell et al. described that FOXC1 is essential for vascular smooth muscle cell differentiation in zebrafish. However, the exact underlying mechanisms of FOXC1 mutation-induced cerebral abnormalities remain unclear [5]. Perivascular spaces are associated with the glymphatic system of the brain [6] and their dilation might reflect systemic abnormalities.

Although neuroradiological abnormalities detected on MRI indicated no progression over several years in several previously described ARS cases, to the best of our knowledge, long-term follow-ups have not been published [7,8]. CSVD is an important cause of stroke, and certain studies suggested that ARS might increase the risk of stroke in affected patients [2,4]. Preventing neurovascular diseases might thus be particularly important during the consultation and management of patients with ARS.

## Patient consent

Informed consent to include the patient’s information in the publication of this case report was obtained.
